# Interocular asymmetry of foveal avascular zone morphology and parafoveal capillary density in sickle cell retinopathy

**DOI:** 10.1371/journal.pone.0234151

**Published:** 2020-06-10

**Authors:** Davis B. Zhou, Adrienne W. Scott, Marguerite O. Linz, Ian C. Han, Maria V. Castanos, Giselle Lynch, Jorge S. Andrade Romo, Rachel E. Linderman, Joseph Carroll, Richard B. Rosen, Toco Y. Chui

**Affiliations:** 1 Ophthalmology, New York Eye and Ear Infirmary of Mount Sinai, New York, New York, United States of America; 2 Icahn School of Medicine at Mount Sinai, New York, New York, United States of America; 3 Retina Division, Wilmer Eye Institute, Johns Hopkins University School of Medicine, Baltimore, Maryland, United States of America; 4 Department of Ophthalmology and Visual Sciences, Institute for Vision Research, University of Iowa, Iowa City, Iowa, United States of America; 5 Cell Biology, Neurobiology & Anatomy, Medical College of Wisconsin, Milwaukee, Wisconsin, United States of America; 6 Ophthalmology & Visual Sciences, Medical College of Wisconsin, Milwaukee, Wisconsin, United States of America; Massachusetts Eye & Ear Infirmary, Harvard Medical School, UNITED STATES

## Abstract

**Objectives:**

To examine interocular asymmetry of foveal avascular zone (FAZ) and parafoveal capillary density metrics in sickle cell retinopathy (SCR) using optical coherence tomography angiography (OCT-A).

**Methods:**

This cross-sectional, retrospective study evaluated SCR patients and unaffected controls who underwent 3x3mm macular OCT-A imaging using a spectral domain-OCT system. FAZ (area, perimeter, and acircularity index) and parafoveal capillary density metrics were computed for both eyes of each participant. In unaffected controls, interocular difference in FAZ and parafoveal capillary density metrics were evaluated using Bland-Altman plots. SCR patients with interocular difference outside the upper 97.5% and lower 2.5% limits of agreement from controls were defined as having interocular asymmetry. Area under receiver operating characteristic curve (AROC) was also performed to determine the ability of the absolute interocular difference to differentiate between subjects with SCR—including non-proliferative SCR (NP-SCR) and proliferative SCR (P-SCR)–and unaffected controls.

**Results:**

Thirty-one patients with SCR (21 NP-SCR and 10 P-SCR) and 14 race-matched and age-matched controls were included for analysis. Interocular asymmetry was seen for all FAZ and parafoveal capillary density metrics in NP-SCR and P-SCR subjects. SCR subjects showed greater disease severity in the left-eye for FAZ and parafoveal capillary density metrics.

**Conclusions:**

NP-SCR and P-SCR patients demonstrated quantifiable interocular asymmetry in FAZ and parafoveal capillary density metrics compared to unaffected subjects, with left-eye predominance in disease severity.

## Introduction

Sickle cell disease is an autosomal recessive hemoglobinopathy that affects nearly 100,000 people in the US [1]. Disease pathophysiology involves misfolding of the beta subunit of hemoglobin, a protein responsible for intravascular oxygen transport. Modified beta-hemoglobin contains exposed hydrophobic regions that facilitate protein polymerization and subsequent erythrocyte deformation, particularly under conditions of cellular stress and deoxygenation [2]. Resulting changes to erythrocyte structure and elasticity impact intercellular processes that mediate inflammation, oxidative injury, endothelial adhesion, and nitric oxide metabolism [3]. These mechanisms increase the risk of vaso-occlusive episodes at various circulatory pathways. This leads to high phenotypic variability in both organ involvement and disease burden [2].

Sickle cell retinopathy (SCR) is the most common ophthalmic sequelae of sickle cell disease [4]. Vascular trauma from the above-mentioned metabolic cascades and recurrent vaso-occlusion leads to retinal ischemia and vision-threatening neovascularization. While studies have traditionally focused on peripheral retinal involvement, vascular changes have also been visualized in the macula: hairpin loops, microaneurysms, parafoveal nonperfusion, and foveal avascular zone (FAZ) modification [5–7]. Intravenous fluorescein angiography (IV-FA) has been the gold standard for identifying vascular pathology and staging disease [4, 8]. It has limitations, however, in spatial resolution and visualization of deep capillary networks. Optical coherence tomography angiography (OCT-A) is an emerging modality that allows dye-free imaging of retinal microvasculature, based on the contrast between erythrocyte movement compared to static surrounding tissue [9, 10]. It offers improved depiction of retinal microvascular networks and reveals quantifiable perfusion changes [4, 11].

Past studies have suggested that SCR manifests asymmetrically between eyes, with descriptions of unilateral involvement [12–14]. This type of interocular asymmetry has been documented in other conditions such as glaucoma and diabetic retinopathy [15, 16], for which relationships to asymmetric upstream blood flow has been reported [17, 18], However, to the best of our knowledge, no studies have quantitatively compared the severity of vascular trauma between eyes in SCR. In this study, we examined interocular asymmetry of FAZ and parafoveal capillary density metrics in SCR patients and unaffected controls using OCT-A [19, 20].

## Materials and methods

### Subjects

This cross-sectional, retrospective study was conducted at the Johns Hopkins School of Medicine Wilmer Eye Institute with data analysis completed at the New York Eye and Ear Infirmary of Mount Sinai. Study protocol was approved by the Institutional Review Board of Johns Hopkins School of Medicine following the Declaration of Helsinki. Patients with a verified diagnosis of sickle cell disease who underwent detailed retinal exam between September 2016 and May 2018 at the Johns Hopkins Wilmer Eye Institute by one author (AWS) were included. Patients received a comprehensive ophthalmic examination that included Early Treatment Diabetic Retinopathy (ETDRS) visual acuity testing, slit lamp and indirect dilated fundoscopy, spectral-domain optical coherence tomography (OCT), and OCT-A for both eyes. Each eye was subsequently staged based on fundus examination, fundus photography, and/or IV-FA according to the Goldberg classification: Stage I = peripheral arterial occlusion; Stage II = peripheral arterio-venous anastomoses; Stage III = neovascular and fibrous proliferation; Stage IV = vitreous hemorrhage; Stage V = retinal detachment [21, 22].

Inclusion criteria were phakic status, clear ocular media, past OCT-A scans for both eyes, and best corrected visual acuity (BCVA) of 20/80 or better. Exclusion criteria included corneal opacification, prior refractive surgery or cataract surgery, anterior segment pathology, dense cataract (grade ≥ 3), macular edema, nystagmus, diabetes, glaucoma, and history of retinopathy from other causes (hypertension, retinal vein occlusion, or HIV). Sickle cell disease patients were then divided into two groups based on the presence of neovascularization in at least one eye: 1) non-proliferative SCR (NP-SCR) group included patients with Goldberg Stage II or below, and 2) proliferative SCR (P-SCR) group consisted of patients with Stage III or above. Unaffected controls—matched according to race and age without a diagnosis of sickle cell disease or other vasculopathy—were also enrolled following the same inclusion and exclusion criteria.

### OCT-A image acquisition

OCT-A scans of the parafoveal vascular network were obtained using a spectral-domain OCT device (Avanti RTVue-XR; Optovue, Fremont, CA, USA; Angioanalytics^™^, version 2018.0.0.16) [23]. Single 3x3mm OCT-A scans were acquired in both eyes of SCR patients and unaffected controls. Scan dimensions in millimeters were computed using the device-default axial length of 23.95mm [24].

### OCT-A image processing and analysis

Image processing was conducted at the New York Eye and Ear Infirmary of Mount Sinai. Scans were evaluated for image quality, with exclusions based on ocular surface drying, blink artefacts, blood vessel doubling, and significant eye motion during acquisition. Analysis was performed using the full retinal vascular slab: from the inner limiting membrane to 70 μm below the posterior boundary of the inner plexiform layer. First, an FAZ mask was created using manually-delineated FAZ borders on Adobe Photoshop CS6 (Adobe Systems, Inc., San Jose, CA, USA). FAZ area, perimeter, and acircularity index were subsequently calculated using MATLAB (The MathWorks Inc., Natick, MA, USA; version 2013a) [25]. Next, noncapillary blood vessels were removed through global thresholding described in prior publications and parafoveal capillaries were extracted from the OCT-A full vascular slab [26, 27]. A parafoveal capillary density map was then created based solely on the extracted capillaries.

Parafoveal capillary density was examined at 5 regions of interest (ROI): over the entire image and at four equiangular quadrants based on the centroid of the FAZ mask (superior, inferior, nasal, and temporal). Global parafoveal capillary density was calculated based on the entire image. For quantitative assessment, parafoveal capillary density was computed for each ROI as described below:

$$Parafoveal\;Capillary\;Density,\;\%=\;\frac{Parafoveal\;Capillary\;Area}{ROI\;area-Noncapillary\;blood\;vessel\;area-FAZ\;area}\;\times 100\%$$

Parafoveal capillary density maps of each SCR patient and unaffected control were then compared to an aged-matched and eccentricity-matched parafoveal-capillary-density normative database published previously (n = 261, age range of 5–87 years, mean of 37 years) [20]. Areas of each scan with low percentiles of capillary density were marked on a deviation map, with regions of parafoveal capillary density below 5% and 1% of the normal distribution highlighted respectively in yellow and red. Subsequently, the percent area highlighted in yellow (below 5%) and red (below 1%) were calculated for each ROI according to the following formula:

$$Percent\;Yellow\;or\;Red\;Area,\%=\;\frac{Yellow\;or\;Red\;Area}{ROI\;area-Noncapillary\;blood\;vessel\;area-FAZ\;area}\;\times 100\%$$

Interocular difference was then calculated for FAZ and parafoveal capillary density metrics so that data from both eyes were incorporated into a single measurement for each patient for further statistical analyses:

$$Interocular\;Difference_{Metric}=OD_{Metric}-OS_{Metric}$$

### Statistical analysis

Analysis was conducted in R (R Foundation for Statistical Computing, Vienna, Austria). Interocular differences in FAZ and parafoveal capillary density metrics of unaffected participants were first examined using Bland-Altman plots [28]. Limits of agreement (LOA)–at the 2.5th percentile and 97.5th percentile—were calculated for each metric in controls. In SCR patients, FAZ and capillary density metrics outside of these limits were defined as exhibiting interocular asymmetry. The frequency and laterality of subjects with interocular asymmetry were then determined for each metric.

Area under receiver operating characteristic curve (AROC) was also performed to determine the diagnostic ability of the absolute value of interocular difference in FAZ and parafoveal capillary density metrics to differentiate participants with SCR (either NP-SCR or P-SCR) from unaffected controls.

## Results

### Demographics

A total of 36 patients with SCR and 20 race-matched and age-matched unaffected controls were included in the study. All subjects were of African-American decent. Five SCR patients and 6 unaffected controls were excluded due to poor OCT-A scan quality or previously-mentioned image artefacts, resulting in 31 SCR patients (21 NP-SCR and 10 P-SCR) and 14 unaffected controls included for analysis. A summary of participant characteristics is shown in Table 1. There were no SCR patients with Stage V disease. Sickle cell disease genotypes included 23 patients with SS or Sβ^0^ thalassemia and 8 with SC or Sβ^+^ thalassemia (Table 1). The percentage of female subjects were 50%, 71.4%, and 64.2% respectively for NP-SCR, P-SCR, and unaffected controls. The mean BCVA were 20/21.2, 20/21.7, and 20/18.7 respectively for NP-SCR, P-SCR, and controls. The median age and range of NP-SCR, P-SCR, and unaffected controls were 28 years (range: 19–51), 39 years (range: 25–57), and 32 years (range: 22–46) respectively. Since not all subject subgroups demonstrated normality, Kruskal-Wallis analysis was performed to assess differences in age between groups and did not reveal significance (P = 0.07).

**Table 1:** Subject characteristics.

Characteristic	Result
Number of subjects included	45
Race	African American
Age–median and range (year)	31 (19–57)
Female–number (%)	29 (64%)
Number of controls	14
Number of patients with sickle cell disease	31
• Stratified by proliferative disease–number (%)	
NP-SCR* (Goldberg Stage ≤ II)	21 (67.7%)
P-SCR* Present (Goldberg Stage ≥ III)	10(32.3%)
• Stratified by genotype	
SS and Sβ^0^thalassemia	23 (74.2%)
SC and Sβ+thalassemia	8(25.8%)
• Prior Treatment	
Hydroxyurea	11 (35.5%)
Oral Anticoagulants	4 (12.9%)
Red Blood Cell Exchange Transfusion	7 (22.6%)
Intraocular Anti-VEGF	0 (0.0%)

Stratification by proliferative disease was based on the eye with more severe pathology

### FAZ metrics

**Fig 1:**
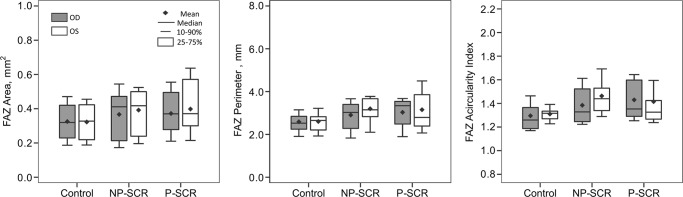
Boxplots of FAZ metrics for the right and left eyes of NP-SCR, P-SCR, and unaffected controls.

**Fig 2:**
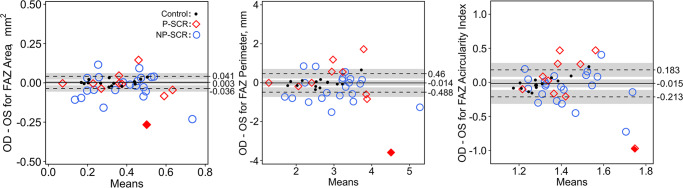
Interocular difference of FAZ metrics characterized by the Bland-Altman analysis. The mean bias (displayed as the solid line) and LOAs (of 2.5% and 97.5% shown in dashed lines) were computed based on unaffected controls, and interocular asymmetry was defined as a value outside these LOAs. The 95% confidence intervals for the mean bias, upper LOA, and lower LOA are shaded in gray. Representative subject labeled with a solid red diamond is displayed in Fig 5.

When grouped by laterality of the eye imaged, no statistically significant difference was found between the left and right eyes for FAZ area, perimeter, or acircularity index in any patient subgroup (Fig 1). Interocular difference then was calculated for unaffected controls in FAZ area, perimeter, and acircularity index–yielding respective upper LOAs of 0.042 mm^2^, 0.46 mm, and 0.18 and lower LOAs of -0.036 mm^2^, -0.49 mm, and -0.21 on Bland-Altman tests (Fig 2). Interocular asymmetry–defined based on the upper and lower LOAs in controls–was subsequently determined all FAZ metrics. NP-SCR patients showed asymmetry in 52.4% of subjects for FAZ area (right-eye: 9.5% versus left-eye: 42.9% severity), 61.9% for FAZ perimeter (right-eye: 14.3% versus left-eye: 47.6% severity), and 33.3% for FAZ acircularity index (right-eye: 9.5% versus left-eye: 23.8% severity). P-SCR subjects exhibited interocular asymmetry in 60.0% of subjects for FAZ area (right-eye: 20.0% versus left-eye: 40.0% severity), 70.0% for FAZ perimeter (right-eye: 40.0% versus left-eye: 30.0% severity), and 50.0% for FAZ acircularity index (right-eye: 40.0% versus left-eye: 10.0% severity). Unaffected controls demonstrated interocular asymmetry in 0% for FAZ area, 7% for FAZ perimeter (right-eye: 7% severity), and 7% of FAZ acircularity index (right-eye: 7% severity).

### Parafoveal Capillary Density Metrics

**Fig 3:**
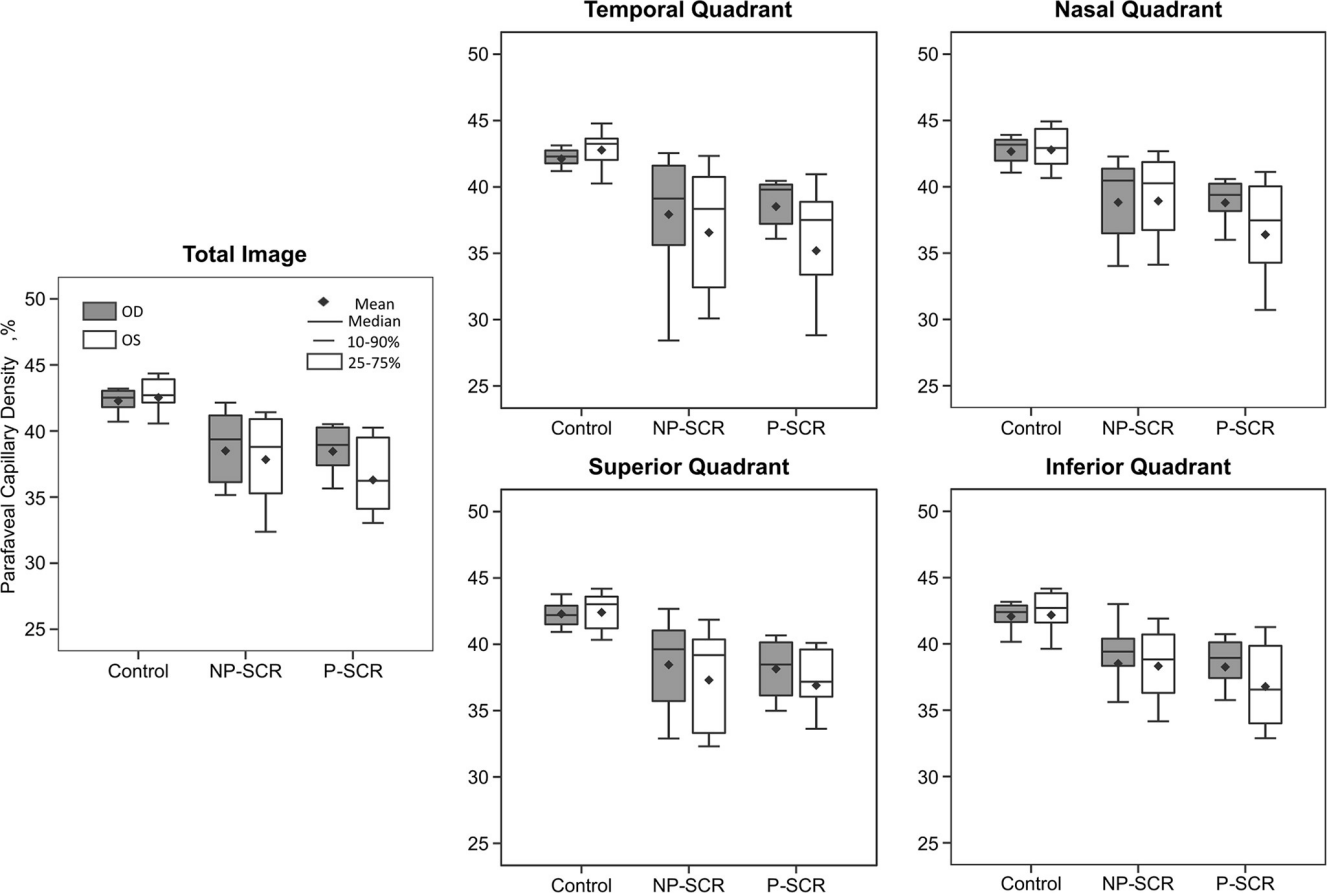
Boxplots of parafoveal capillary density measurements for the right and left eyes of NP-SCR, P-SCR, and unaffected controls.

**Fig 4:**
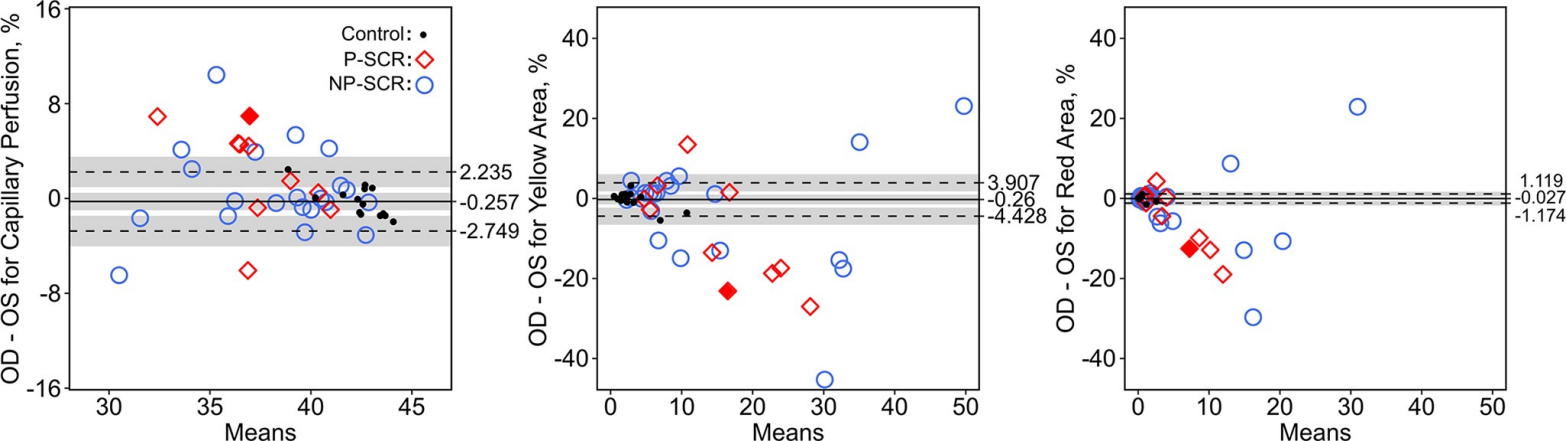
Interocular difference of parafoveal capillary density measurements following Bland-Altman analysis. The mean bias (displayed as the solid line) and LOAs (of 2.5% and 97.5% shown in dashed lines) were computed based on unaffected controls, and interocular asymmetry was defined as a value outside these LOAs. The 95% confidence intervals for the mean bias, upper LOA, and lower LOA are shown in gray. Representative subject labeled with a solid red diamond is displayed in Fig 5.

Direct comparison of mean global parafoveal capillary density between the right and left eyes revealed increasing differences from unaffected controls (42.3% versus 42.5%) to NP-SCR (38.5% versus 37.8%) and P-SCR subjects (38.5% versus 36.3%), with boxplots shown in Fig 3. Bland-Altman analysis revealed interocular differences in global parafoveal capillary density, percent yellow area, and percent red area for controls–with respective upper LOAs of 2.2%, 3.9%, and 1.1% and lower LOAs of -2.7%, -4.4%, and -1.2% (Fig 4). N-PSCR participants demonstrated interocular asymmetry in 42.86% of patients for global parafoveal capillary density (right-eye: 14.29% versus left-eye: 28.57% severity), 52.38% for percent yellow area (right-eye: 23.81% versus left-eye: 28.57% severity), and 42.86% for percent red area (right-eye: 14.29% versus left-eye: 28.57% severity). P-SCR patients revealed asymmetry in 60% of patients for global parafoveal capillary density, percent yellow area, and percent red area (right-eye: 10% versus left-eye: 50% severity for all global parafoveal capillary density metrics). Control subjects exhibited interocular asymmetry in 7% for global parafoveal capillary density, percent yellow area, and percent red area (left-eye: 7% severity for all global parafoveal capillary density metrics).

### Area under receiver operating characteristic curve

AROC analysis was used to compare the ability of absolute interocular asymmetry in FAZ and parafoveal capillary density metrics to differentiate patients with SCR from unaffected controls (Table 2). Among FAZ metrics, interocular asymmetry in FAZ area displayed the highest predictability (AROC = 0.82), followed by FAZ perimeter (AROC = 0.78), and FAZ acircularity index (AROC = 0.76). Interocular asymmetry in parafoveal capillary density displayed poor diagnostic value at all ROI (AROC ranging from 0.62 to 0.70). Analysis of percent yellow and percent red areas demonstrated good differentiative ability using the global parafovea, with AROC = 0.81 and AROC = 0.82, respectively. Good diagnostic ability was also seen on quadrant analysis, with highest differentiation using the percent red area of the nasal quadrant (AROC = 0.87).

**Table 2:** Area under receiver operating characteristic curve.

Metrics		AROC (95% Confidence Interval)
FAZ	Area	0.82 (0.70–0.94)
	Perimeter	0.78 (0.65–0.92)
	Acircularity Index	0.76 (0.62–0.91)
Parafoveal Capillary Density	Entire Image	0.68 (0.52–0.83)
	Temporal	0.63 (0.46–0.79)
	Nasal	0.71 (0.56–0.86)
	Superior	0.70 (0.54–0.85)
	Inferior	0.69 (0.53–0.85)
Percent Yellow Area	Entire Image	0.81 (0.68–0.94)
	Temporal	0.74 (0.59–0.88)
	Nasal	0.83 (0.71–0.96)
	Superior	0.82 (0.69–0.96)
	Inferior	0.81 (0.68–0.94)
Percent Red Area	Entire Image	0.82 (0.70–0.95)
	Temporal	0.84 (0.73–0.96)
	Nasal	0.87 (0.76–0.99)
	Superior	0.83 (0.71–0.95)
	Inferior	0.79 (0.64–0.93)

Area under the receiver operating curve (AROC) analysis for discriminating between patients with SCR (NP-SCR or P-SCR) and unaffected controls based on the absolute value of interocular asymmetry in FAZ and parafoveal capillary density metrics.

## Discussion

Interocular asymmetry in SCR has been suggested by previous studies examining unilateral manifestation of retinopathy. Jampol et al. reported unilateral involvement in 63% of P-SCR subjects enrolled in a prospective clinical trial [13]. Unilateral manifestation was also seen in 61% percent of P-SCR patients of a prospective study involving 407 children with sickle cell disease [12]. Differences in laterality of Stage III P-SCR have also been documented, with 67 unilateral versus 3 bilateral cases found among 101 patients [14]. Subjects were evaluated using biomicroscopy, fundus photography, and IV-FA in each of these above-mentioned studies. Presence of SCR defined by OCT retinal thinning also demonstrated unilateral disease manifestation in 31% of SCR patients [29]. Our findings of interocular asymmetry in 60% of P-SCR patients for global parafoveal capillary density, percent yellow area, and percent red area is consistent with these previously-described rates of unilateral involvement on fundus photography and IV-FA. Our study furthermore characterized asymmetry in NP-SCR patients and examined the laterality of interocular asymmetry in both NP-SCR and P-SCR patients.

**Fig 5:**
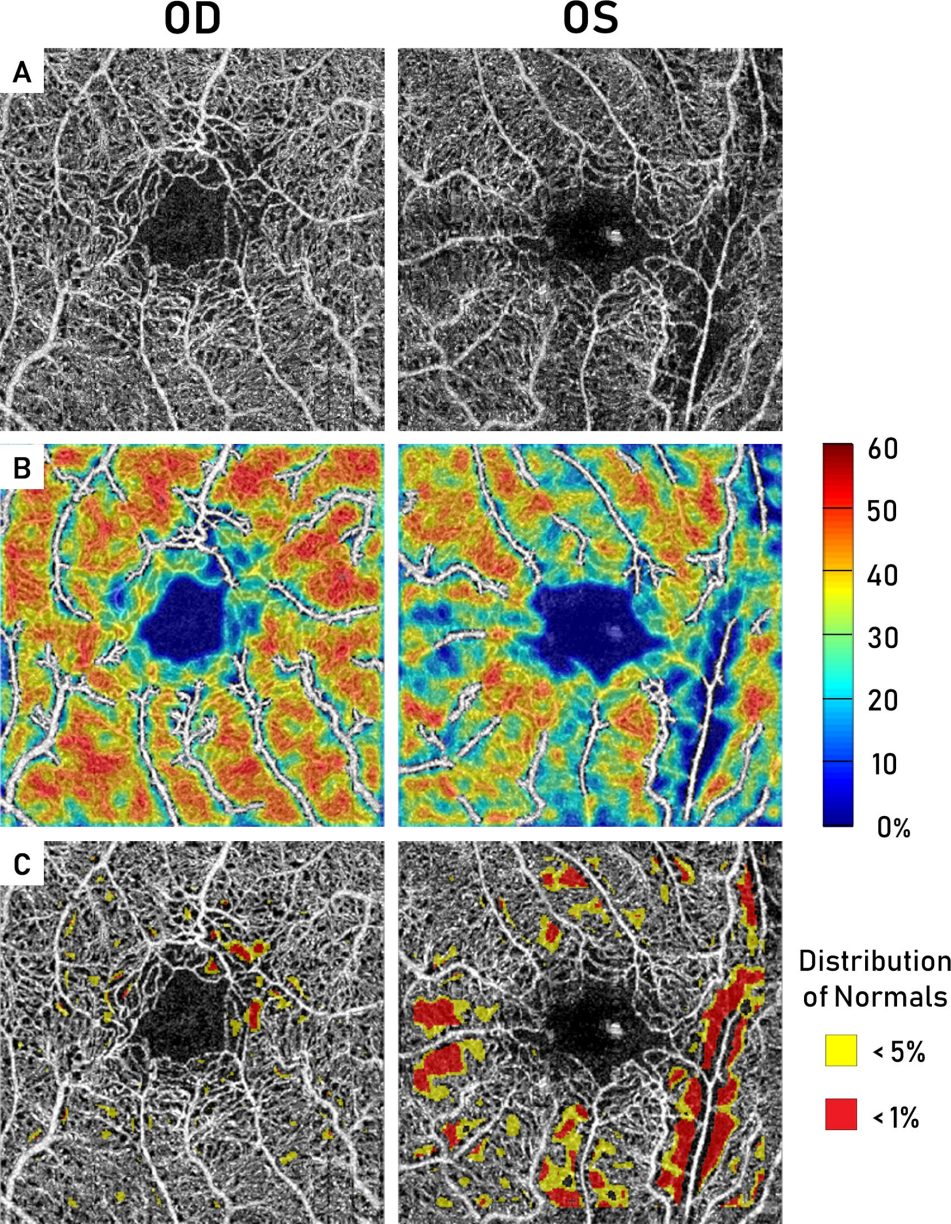
Representative eyes demonstrating interocular asymmetry in a P-SCR patient. Asymmetry is seen between eyes on the A) OCT-A full vascular retinal slabs, B) Parafoveal capillary density maps, and C) Parafoveal capillary density deviation map analysis.

Interocular asymmetry was determined for FAZ and parafoveal capillary density metrics. The LOAs encompassing the 95% distribution of unaffected controls were used to define the threshold for interocular asymmetry in SCR patients for each metric. A representative patient with such asymmetry is illustrated in Fig 5. Greater parafoveal capillary nonperfusion is seen in the left eye compared to the right. This decrease in perfusion is particularly evident in the temporal parafovea of the more severe eye, which is consistent with previous studies [19, 30]. Greater asymmetry was seen in P-SCR than in NP-SCR patients for all FAZ and global parafoveal capillary density metrics, suggesting that asymmetric differences in disease severity seen earlier in the disease course may continue to progress asymmetrically.

In these patient groups, the laterality of asymmetry suggested greater disease severity in the left eye for FAZ and parafoveal capillary density metrics in both NP-SCR and P-SCR subjects. Although these findings may be simply due to chance in a relatively small sample, the consistent laterality suggests the presence of anatomical or physiological factors that contribute to greater disease progression in the left eye. This may include anatomical differences between the right and left carotid arteries that supply the ophthalmic arteries. The left common carotid artery branches directly from the aorta while the right common carotid emerges from the brachiocephalic branch of the aorta. Past studies have revealed distinctions between the right and left carotid arteries in their response to stressors such as hypertension, which is hypothesized to involve differences in blood flow hydrodynamics between the two sides [31]. Such variations could affect downstream circulation, which is substantiated by the higher incidence of left-sided strokes in the middle cerebral artery (MCA)–the vessel directly distal to the internal carotid artery [32, 33]. Asymmetry in local blood flow in the MCA is has furthermore been documented in sickle cell patients compared to unaffected controls [34]. The ophthalmic artery—as a distal branch of the internal carotid artery—and may experience similar influences that impact downstream retinal circulation. Previous studies have found relationships between carotid flow and asymmetric manifestations of other ophthalmic conditions, including glaucoma and diabetic retinopathy [17, 18]. In SCR, differences in carotid flow and downstream ophthalmic circulation may facilitate left-sided preference of ischemic or vaso-occlusive events that lead to FAZ and capillary density changes.

There were several limitations to this study. The small sample size reduced the ability to examine differences in interocular asymmetry between all five stages of SCR. Image processing was also conducted on the full retinal vascular slab in order to reduce confounding effects from OCT segmentation and OCT-A projection artefacts. Analysis with the merged superficial and deep parafoveal capillary plexus, however, prevented the ability to distinguish contributions from each individual capillary network. OCT-A imaging also exhibits limited ability to identify vessels with velocity below the system detection threshold. This limitation may have a greater influence for SCR patients, as reduced flow may be more prevalent due to erythrocyte aggregation and adhesion to vessel endothelium [3]. These areas of decreased flow would be measured as non-perfused capillaries on OCT-A, leading to an overestimation of changes in FAZ and parafoveal capillary density metrics. In addition, this study did not examine other factors that may contribute to asymmetry of imaging metrics, including differences in axial lengths or refractive error between eyes. The use of device-default axial length may influence raw FAZ and parafoveal capillary density metrics.

## Conclusion

In summary, patients with NP-SCR and P-SCR demonstrated quantifiable interocular asymmetry in FAZ and parafoveal capillary density metrics compared to unaffected controls. Laterality of asymmetry suggested greater disease progression in the left eyes of patients, and the frequency of asymmetry appears to increase with greater disease severity. Interocular asymmetry in parafoveal capillary density deviation map measurements also demonstrated ability to differentiate SCR from unaffected controls. In these ways, interocular asymmetry of OCT-A findings provide an additional method for characterizing disease in clinical settings. These measurements may provide utility especially in tracking progression of disease due to their quantitative nature and in identifying mild disease as demonstrated by the study population of early stage patients.

## Supporting information

S1 Table Interocular asymmetry analysis percentages. (XLSX)
